# T-cell receptor determinants of response to chemoradiation in locally-advanced HPV16-driven malignancies

**DOI:** 10.3389/fonc.2023.1296948

**Published:** 2024-01-03

**Authors:** Pablo Nenclares, Adrian Larkeryd, Floriana Manodoro, Jen Y. Lee, Susan Lalondrelle, Duncan C. Gilbert, Marco Punta, Ben O’Leary, Antonio Rullan, Anguraj Sadanandam, Benny Chain, Alan Melcher, Kevin J. Harrington, Shreerang A. Bhide

**Affiliations:** ^1^ Radiotherapy and Imaging Division, The Institute of Cancer Research, London, United Kingdom; ^2^ Head and Neck Unit, The Royal Marsden Hospital, London, United Kingdom; ^3^ Bioinformatics Unit, The Centre for Translational Immunotherapy, The Institute of Cancer Research, London, United Kingdom; ^4^ Genomics Facility, The Institute of Cancer Research, London, United Kingdom; ^5^ Sussex Cancer Centre, University Hospitals Sussex NHS Foundation Trust, Brighton, United Kingdom; ^6^ Unit of Immunogenetic, Leukemia Genomics and Immunobiology, IRCCS Ospedale San Raffaele, Milan, Italy; ^7^ Systems and Precision Cancer Medicine Team, The Institute of Cancer Research, London, United Kingdom; ^8^ Division of Infection and Immunity, University College London, London, United Kingdom

**Keywords:** human papillomavirus, radiotherapy, T-cell receptor, cervical cancer, anal cancer, head and neck cancer

## Abstract

**Background:**

The effect of chemoradiation on the anti-cancer immune response is being increasingly acknowledged; however, its clinical implications in treatment responses are yet to be fully understood. Human papillomavirus (HPV)-driven malignancies express viral oncogenic proteins which may serve as tumor-specific antigens and represent ideal candidates for monitoring the peripheral T-cell receptor (TCR) changes secondary to chemoradiotherapy (CRT).

**Methods:**

We performed intra-tumoral and pre- and post-treatment peripheral TCR sequencing in a cohort of patients with locally-advanced HPV16-positive cancers treated with CRT. An in silico computational pipeline was used to cluster TCR repertoire based on epitope-specificity and to predict affinity between these clusters and HPV16-derived epitopes.

**Results:**

Intra-tumoral repertoire diversity, intra-tumoral and post-treatment peripheral CDR3β similarity clustering were predictive of response. In responders, CRT triggered an increase peripheral TCR clonality and clonal relatedness. Post-treatment expansion of baseline peripheral dominant TCRs was associated with response. Responders showed more baseline clustered structures of TCRs maintained post-treatment and displayed significantly more maintained clustered structures. When applying clustering by TCR-specificity methods, responders displayed a higher proportion of intra-tumoral TCRs predicted to recognise HPV16 peptides.

**Conclusions:**

Baseline TCR characteristics and changes in the peripheral T-cell clones triggered by CRT are associated with treatment outcome. Maintenance and boosting of pre-existing clonotypes are key elements of an effective anti-cancer immune response driven by CRT, supporting a paradigm in which the immune system plays a central role in the success of CRT in current standard-of-care protocols.

## 1 Introduction

Chemoradiotherapy (CRT) is the cornerstone of treatment for locally-advanced malignancies related to human papillomavirus (HPV) infection, including head and neck squamous cell (HNSCC), anal squamous cell (ASCC) and cervical carcinomas (CC). Despite being a highly effective therapy for most patients, around 15 to 25% of those diagnosed with locoregionally advanced disease will relapse within 5 years of treatment (1–3). Increasing evidence suggests that CRT plays a role in the anti-cancer immune response, promoting a number of anti-tumor immune mechanisms such as improved antigen cross-presentation, increased type I interferon release, enhanced expression of major histocompatibility complex (MHC) class I on tumor cells, recruitment and maturation of dendritic cells, promotion of the infiltration of lymphocytes into the tumor and augmentation of cytotoxic T cell activation (4, 5). However, whether these processes have any clinical relevance in the response to CRT remains unclear. Several studies have investigated intra-tumoral and peripheral adaptive responses to immunotherapy, but there is a dearth of similar studies in the context of CRT (6–11).

T-cell receptor (TCR) sequencing has emerged as a powerful new method in the analysis of the host-tumor interaction, which partially enables the characterization of the dynamics of the adaptive anti-cancer immune response (12, 13). We hypothesized that, since anti-cancer immune responses, including those promoted by radiotherapy, can mirror normal defensive responses to pathogens, it might be possible to study patient responses to CRT by monitoring peripheral T-cell clonal dynamics during treatment (14). In this study, we used HPV16-driven cancers diagnosed at a locally-advanced stage and treated with curative-intent standard-of-care CRT as a model for investigating TCR metrics and dynamics that may be linked with patient response.

HPV-related malignancies are distinguished by the expression of viral oncoproteins, such as E2, E5, E6 and E7, which can serve as tumor-specific antigens (15–18). Within the different high-risk HPV subtypes, HPV16 is the most frequently associated with oropharyngeal (85%), cervical (55%) and anal carcinomas (90%) (19–21). Given the favorable prognosis associated with HPV-positive HNSCC, the concept of de-escalation strategies has been developed to reduce treatment-related long-term toxicities whilst maintaining excellent survival outcomes (22). However, there is mounting evidence that HPV-positive tumors are not uniform and that differences in the phenotype and immune response between distinct HPV-driven tumors subtypes have an impact on prognosis and survival, suggesting that different treatment approaches might be required (23, 24). Taking this into consideration, anti-PD-1/PDL-1 based therapies and therapeutic HPV vaccination in the neoadjuvant and adjuvant setting for HPV-mediated cancers have attracted research attention with the aim of improving responses to CRT (25). Nevertheless, published results of Phase III randomized trials tell a cautionary tale of treatment de-escalation, and negative results from Phase III randomized trials of anti-PD-1/PDL-1 based therapies in the concurrent and/or adjuvant setting, ultimately highlight the importance of further investigation into the effect of CRT on antigen-specific immune responses (26, 27).

In the current study, we set out to sequence the T-cell complementarity-determining region 3 of the β-chain (CDR3β) in both the pre-treatment tumor-infiltrating lymphocytes (TILs) and pre- and post-treatment peripheral blood mononuclear cells (PBMCs) among a cohort of patients with HPV-related cancers treated with standard-of-care radical CRT. We characterized the diversity, clonality and degree of sequence similarity of the TCR repertoire and looked for associations between different metrics and response to CRT. In addition, we performed in silico antigen-specificity clustering and bioinformatically tested for affinity between HPV16-derived antigens predicted to be presented according to the individual MHC complex of each patient and these clusters. Overall, the approach of TCR profiling presented here enables a comprehensive analysis of the T-cell repertoire and the identification of some relevant determinants of response to CRT. Our results identify several repertoire features which associate the response to therapy. These features provide further biological understanding of the anti-cancer T-cell immune responses in HPV-driven malignancies and may lead to improved selection of patients for de-escalation strategies or intensification with adjuvant immunotherapy.

## 2 Materials and methods

### 2.1 Study design

CCR4157 is a single-arm, translational, sample collection study of standard-of-care CRT in locally-advanced HPV-positive malignancies including HNSCC, ASCC and CC. Informed consent was obtained from all eligible patients with stage III/IVB HNSCC, stage II/IIIB (AJCC 2007) ASCC and stage IIB/IVA (FIGO) CC. Institutional board and ethics committee (ref. no. 14/NE/1055) approved the study. In all cases, radiotherapy was delivered using simultaneous integrated boost-IMRT technique. All HNSCC patients were treated with doses of 65 Gy in 30 fractions (2.17 Gy/fraction) to the primary target and doses to the elective target were 54 Gy in 30 fractions (1.8 Gy/fraction) over 6 weeks. All HNSCC patients received concurrent cisplatin 100 mg/m^2^ days 1 and 29). Radiotherapy for CC patients included external beam radiotherapy (total dose of 45 Gy in 28 fractions and boost to the involved nodes to total dose of 55-57 Gy) followed by intrauterine brachytherapy treatment to a total combined dose > 85 Gy EQD2. CC patients received weekly cisplatin (40 mg/m^2^). Radiotherapy for the ASCC cohort was planned as per UK IMRT anal cancer guidelines and prescribed to a total dose of 53.2 Gy (50.4 Gy for stage II disease) to the primary disease, 50.4 Gy to the affected lymph nodes and 40 Gy to elective volume, administered in 28 days over 38 days. All patients diagnosed with ASCC received concurrent mitomycin C 12 mg/m^2^ on day 1 and oral capecitabine 825 mg/m^2^ BID on each radiotherapy day. At 12 weeks, response following treatment was assessed by clinical examination and ^18^F-FDG PET-CT. Residual or equivocal uptake at primary and distant sites was confirmed with a biopsy.

Patients were classified as responders if they achieved a complete response (CR) in ^18^F-FDG PET-CT 12 weeks following completion of radical CRT and there was no evidence of relapse within the next 5 years after treatment. Patients were considered non-responders if the ^18^F-FDG PET-CT showed evidence of local and/or regional residual disease or progressive disease (which was subsequently histologically confirmed) and/or the patient showed histologically confirmed evidence of local, regional or distant relapse within the first year after completion of radical CRT. Patient with local, regional, or distant relapse within 2 to 5 years following completion of CRT were excluded from this sub-analysis.

Sample estimation was based on the hypothesis that both groups (responders and non-responders) would present a standard deviation (SD) in the clonality (1-normalised Shannon Index) of 0.4 and that the study would be able to detect a difference of 0.6 in the mean clonality values. This assumption was based on internal (not previously published) preliminary data of a small cohort of patients not included in this study. The required sample size based on these values was 8 patients for each group with a confidence level of 95%, a power of 80% and a two-sided contrast.

### 2.2 Sample collection

Tumor tissue was collected via biopsy at baseline. Serial blood samples were collected at baseline (pre-CRT), 6 weeks and 12 weeks following completion of CRT. Twenty milliliters of blood collected in Streck^®^ tubes was centrifuge at 1,600 rpm for 10 min within 3 hours of collection and plasma and buffy coats were isolated and kept frozen at -80°C prior to DNA extraction.

### 2.3 Nucleic acid extraction

Five 10µm unstained slides and one hematoxylin and eosin-stained slides were obtained from representative formalin fixed paraffin embedded (FFPE) tumor blocks. Experienced pathologists assessed tumor content and suitable areas of tumor were marked for microdissection, if necessary. RNA and DNA were extracted from FFPE tumor blocks using AllPrep DNA/RNA FFPE kit (Qiagen). DNA from peripheral blood mononuclear cells (PBMC) was extracted using QIAamp DNA Blood mini kit (Qiagen) from the buffy coats. Nucleic acid yield and quality were assessed on Qubit Fluorometric quantification (ThermoFisher Scientific) and TapeStation4200 (Agilent).

### 2.4 HPV detection in tumor, TCR sequencing and HLA typing

P16 ^INK4A^ status of tumors was confirmed using immunohistochemistry and all immunohistochemical interpretations were made by consultant histopathologists. Diffuse strong nuclear expression of >70% of tumor nuclei for p16 was considered positive. RNA extracted from FFPE blocks was used for cDNA synthesis using the High-Capacity cDNA Reverse Transcriptase kit (Thermo Fisher). Evidence of HPV16 integration was assessed by detection of E7 expression using methods and primers described previously in a 7500 Sequence detection system (Applied Biosystems, Foster City, CA, USA/Thermo Fisher) (28). Any specimen producing dCt< 13 was consider positive for HPV integration. Detection of HPV16 DNA in tumor was performed using a “HPV16-detect” novel NGS assay with Ion AmpliSeq Designer (ThermoFisher Scientific) as previously described (29).

TCR β-chain sequencing was performed utilizing the genomic DNA extracted from FFPE tumor samples or from buffy coat samples by using Adaptive kit. Sequencing libraries were generated using the ImmunoSEQ kit (Adaptive Biotechnologies) according to the manufacturer’s recommendations. The first round of PCR was carried out using the ImmunoSEQ proprietary PCR primer mix (32 µL per sample containing 25 uL of QIAGEN 2x Multiplex PCR Master Mix, 5µL of QIAGEN 5x Q-solution and 2 µL of primer mix). A positive control reaction, provided in the kit, and a negative control reaction were included with each sample batch. PCR cycling parameters were: heated lid (105°C), 95°C 5 min denaturation step, followed by 21 cycles of 94°C, 30 s denaturation, 65°C, 75 s annealing, and 72°C, 40 s extension; followed by 72°C, 10 min final extension, then hold at 4°C. Amplified libraries were diluted using the DNA suspension buffer (30 µL) provided. A second round of PCR was performed to generate uniquely barcoded sequencing libraries using the barcode primer plate included in the kit (17µL of working mix which include 12.5µL QIAGEN 2x Multiplex PCR master mix, 2.5µL QIAGEN 5x Q-solution and 2µL of QIAGEN RNase-free water; 4 µL of primers from the provided barcode plates and 4 µL of the first PCR product). Second PCR cycling parameters were: heated lid (105°C), 95°C 15 min denaturation step, followed by 21 cycles of 94°C, 30 s denaturation, 68°C, 40 s annealing, and 72°C, 60 s extension; followed by 72°C, 10 min final extension, then hold at 12°C. The quality of the libraries was assessed using Agilent 4200 TapeStation High Sensitivity D1000 Screentape. Samples were pooled volumetrically and purified using MAGBIO HighPrep PCR beads (1x). The final pool was quantitated using a Kapa Library Quantification kit for Illumina. Libraries were sequenced on the Illumina MiSeq System following the manufacturer’s instructions and using MiSeq Reagent Kit v3 (150-cycle) Single-Read. A total of 168 sequencing cycles were performed (Read 1:156 cycles, Read 2: 12 cycles), as recommended in the protocol, adding 5% PhiX. ImmunoSEQ platform was used for TCR identification and CDR3 extraction.

High-resolution HLA typing from genomic DNA extracted from blood was carried out by NGS at VH Bio (UK) for all patients.

### 2.5 TCR repertoire metrics

The clonality index was estimated for each sample by using the command clonality from the LymphoSeq R package (30). This score is derived from the Shannon entropy, which is calculated from the frequencies of all productive CDR3β sequences divided by the logarithm of the total number of productive sequences. This normalized entropy value is then inverted (1-normalised Shannon index) to produce the clonality metric.

Clonal relatedness was estimated using the “clonalRelatedness” command from the LymphoSeq R package with an edit distance of 3. Morisita index as a statistical method for overlap in a population, was calculated using the repOverlap(method=“morisita”) command from the immunarch package in R (31).

Expanded TCR clonotypes either in tumor or in blood were those present above a threshold of relative frequency of 2/1,000 (corresponding to the top 1% of the empirical TCR frequency distribution). At this threshold, which has been already described in previously published work, the correlation between clonality and proportion pf repertoire occupied by expanded TCRs is very strong and the number of TCRs labelled as expanded is greater than for higher thresholds for which this correlation is also significant (11).

The difference in abundance between pre- and post-treatment peripheral blood was calculated with the Fisher test function in LymphoSeq package in R. This function assumes that the repertoire contains *S* distinct clones and their proportional abundances in paired samples (sample 1 and sample 2) are given by the multinomial vectors p^(1)^={p_1_^(1)^, p_2_^(1)^, …, p_S_^(1)^} and p^(2)^={p_1_^(2)^, p_2_^(2)^, …, p_S_^(2)^} with $\sum_{i=1}^{S}p_{i}^{\left(j\right)}$ =1. Supposing that *n* clones change in abundance between the two timepoints, these clones can be identified with the *n* element index vector Δ. Next, assuming that the aggregated change of a truly changed TCR abundance is small [i.e. $\sum_{iЄ\Delta }\left(p_{i}^{\left(2\right)}-p_{i}^{\left(1\right)}\right)$ «1] each observed clone can be independently tested for significance using a two-by-two contingency table and by employing a Fisher exact test to compute the *p* value for each clone across the two timepoints, against the null hypothesis that the abundance of the clone is identical in the two samples. To identify the set of significantly changed clones between the two timepoints, a positive false discovery rate (FDR) method of Storey is used (32, 33). TCRs with FDR *q*-values< 0.01 were labelled as statistically significant expanded or contracted.

### 2.6 CDR3β amino acid clustering

Pairwise similarity between pairs of TCRs was measured on the basis of amino acid triplet sharing. Sharing was quantified using the normalized string kernel function. The kernel is calculated as the number of amino acid triplets (sets of three consecutive amino acids) shared by two CDR3βs, normalized by the number of triplets in each CDR3β being compared. Two TCRs were considered connected if the similarity index was ≥0.7. Per (patient, timepoint) pair, the number of clusters containing an expanded CDR3β was counted.

For the maintained and replaced clustering methods, peripheral baseline expanded clonotypes that were shared with and expanded in tumor were selected and subsequently classified in either maintained or replaced clonotypes. Following this, the clustering was done as explained before using the same similarity index threshold. Per repertoire (maintained or replaced), the number of clonotypes that were part of a cluster at baseline were counted.

### 2.7 In silico antigen specificity clustering pipeline

The GLIPH version 2 algorithm was implemented for the establishment of T-cell specificity groups using the CDR3β sequences from tumor and peripheral blood at each timepoint and the HLA types (34, 35). The parameters used to run GLIPH2 were local minimum p value of 0.001, p depth of 1000, global convergence cutoff of 1, simulation depth of 1000, kmer minimum depth of 3, local minimum OVE of 10, and accepting all amino acids interchangeable. Briefly, by comparing the input with the reference dataset of 273,920 distinct CDR3β sequences (both CD4 and CD8) from 12 healthy individuals, GLIPH2 first discovered clusters of CDR3β sequences sharing either global or local motifs, as previously described (34). Previously used in a large cohort of non-small cell lung cancer (NSCLC) patients, GLIPH2 algorithm has shown to enable the analysis of shared TCR specificity and HLA prediction (35). The output of GLIPH2 include the CDR3β clusters with shared sequence motifs and is accompanied by multiple statistical measurements to facilitate the calling of high-confidence specificity groups, including biases in Vβ gene usage, CDR3β length distribution (only relevant for local motifs), cluster size, HLA allele usage, and clonal expansion.

A scaled count for each patient in each GLIPH2 convergence group containing sequences from more than 10 samples (n=30,222) was used as input to the UMAP. UMAP analysis was performed using the umap R package. The UMAP parameters that were changed from default were n_neighbours (parameter that control how UMAP balances local versus global structure of the data) was set to 302 (the GLIPH2 converge group count divided by 100) and min_dist (parameter that provide the minimum distance apart that points are allowed to be in the low dimensional representation) set to 0.25. Based on the UMAP components, HDBSCAN was used to cluster the GLIPH2 convergence group (R package hdbscan) with min_cluster_size set to 100 (parameter set to smallest size grouping aimed to consider a cluster) and min_samples set to 0 (lowest conservative threshold as possible for clustering, since the input was previously obtained GLIPH2 convergence groups). For each HDBSCAN cluster, the distributions of the scaled counts were compared between responders and non-responders and across timepoints using a Wilcoxon rank sum test and a signed-rank test, respectively. Any clusters with an FDR *q-*value of< 0.01 is shown circled in the graph (it is a three SD ellipse, based on all the points in the cluster). Next, we used netMHCpan-4.1 to select those 8-11 mers derived from the HPV16 oncoproteins E2, E5, E6 and E7 that were binders (percentage elution rank<1) according to the HLA type of each patient (36). We used the UMAP clusters output (CDR3β amino acid sequence) and the netMHCpan-4.1 output as the input to run ERGO pipeline and estimate the TCR-epitope binding probability (37). Finally, binding probabilities for each UMAP cluster was compared against those for all other clusters combined using Fisher exact test. Clusters with FDR *q*-values< 0.01 were labelled as displaying statistically significant higher (if Fisher stat< 1) or lower (of Fisher stat > 1) binding probability compared to the other clusters. GLIPH2, UMAP, HDBSCAN, netMHCpan4.1., ERGO and clustering comparison code are available in github: https://github.com/instituteofcancerresearch/CCR4157.

### 2.8 Quantification and statistical analysis

Statistical analysis was performed in R and GraphPad Prism 8. We used the T-test two-tailed paired or non-paired (or Mann-Whitney non-parametric test as appropriate when normality was not passed using Shapiro-Wilk test) to test for statistical differences in the mean between two samples. We used one-way ANOVA paired or non-paired (or non-parametric tests as matched Friedman test, as appropriate) to compare means between more than two samples, or two-way ANOVA (or mixed model) for grouped analysis. Significant values were corrected for multiple testing using Sidak’s or Dunn’s correction when appropriate. A p value less than 0.05 was considered significant. TCRβ V and J gene usage comparisons were done using Kruskall-Wallis test with Holm method correction. Data visualization was performed in R and GraphPad Prism 8. All graphs show bars with median and 95% confidence interval (CI).

## 3 Results

### 3.1 Cohort overview and patient characteristics

A total of 19 patients were recruited into this sub-study. These included 6 HNSCC, 6 ASCC and 7 CC and patients were classified as responders (n=11) or non-responders (n=8). The median time to relapse for the non-responder group was 192 days following completion of CRT and the average follow-up for the responder group was 5.61 years. Responders included 4 HNSCC, 4 ASCC and 3 CC while non-responders included 2 HNSCC, 2 ASCC and 4 CC (z test p value = 0.6713). Details for all patients are shown in **Table 1**. Baseline tumor biopsies were available for all patients, baseline blood was available for 18 patients and post-treatment (6 and 12 weeks) blood samples were available for 18 and 19 patients, respectively. All patients were confirmed to be positive for p16^INK4A^ and HPV16 E7 in tumor by immunohistochemistry and detection of HPV16 E7 mRNA and HPV16 DNA with “HPV-detect”, respectively. When testing for HPV18, 31 and 33 DNA, no other HPV subtypes were detected.

**Table 1:** Patient characteristics.

	N	%
Age, years, mean (SD)	45.82 (25.16)	-
Sex		
** Male**	6	31.5
** Female**	13	68.5
Type of cancer		
** Head and neck (oropharynx)**	6	31.5
** Anal**	6	31.5
** Cervical**	7	37
Histology		
** Squamous cell carcinoma**	17	89.5
** Adenocarcinoma**	2	10.5
Node status		
** Positive**	12	63
** Negative**	7	37
Stage *		
** I**	3	16
** II**	8	42
** III**	7	37
** IV**	1	5
Response		
** Responders**	11	58
** Non-responders**		
** - PR/PD after CRT**	4	21
** - Early PD (<1 year)**	4	21

*Stage by AJCC TNM 8^th^ Edition for head and neck and anal carcinoma and FIGO 20^th^ Edition for cervical cancer.

CR, complete response; PD, progressive disease; PR, partial response.

### 3.2 CRT increases the peripheral T-cell clonality, which correlates with response to treatment

**Figure 1:**
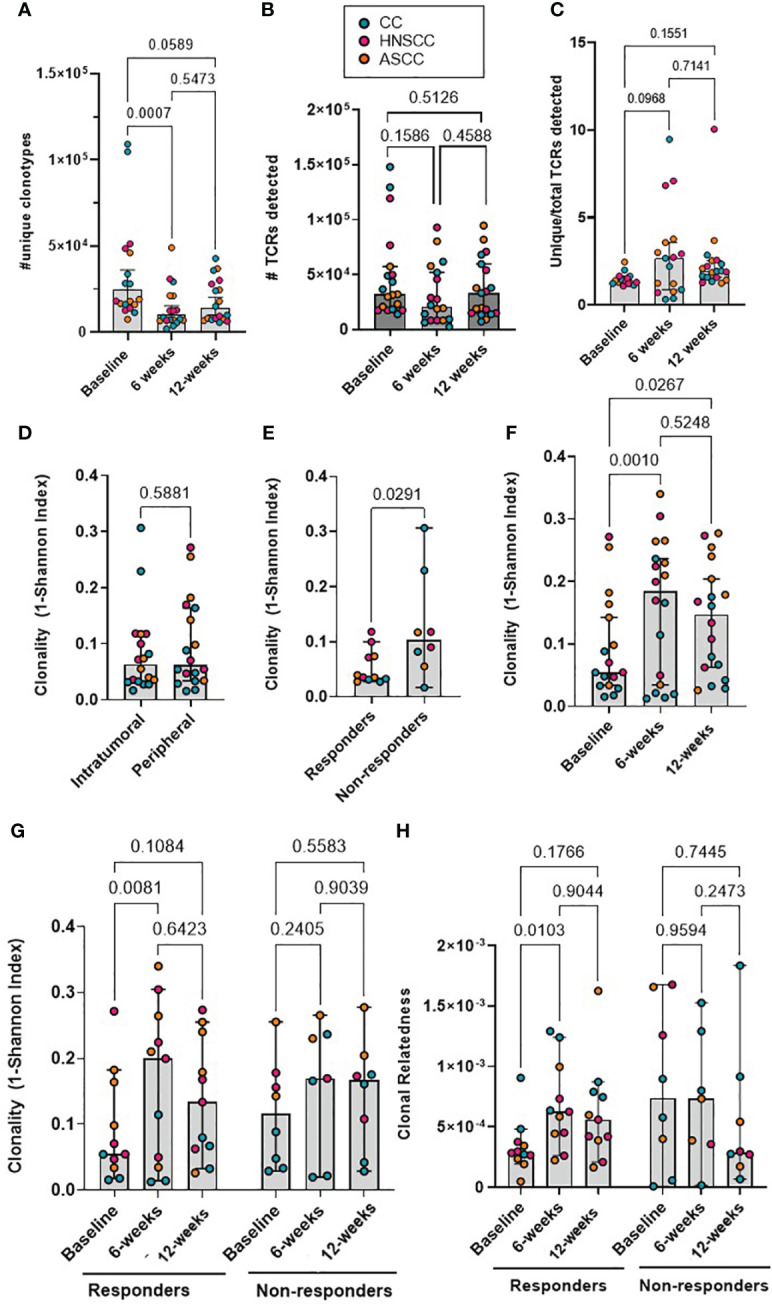
**(A)** The absolute number of unique peripheral TCR clonotypes are shown for each patient at each timepoint. **(B)** Absolute number of TCRs detected for each patient at each timepoint. Friedman test (paired samples) with Dunn’s multiple comparisons p values are shown for figures A and B. **(C)** Proportion of unique clonotypes in relation to total TCRs detected in peripheral repertoire. Mixed-effect analysis with Dunnett’s multiple comparisons p values are shown. **(D)** The intra-tumoral and baseline peripheral TCR repertoire clonality scores are shown for each patient. Paired t test p value shown. **(E)** The intra-tumoral TCR repertoire clonality scores are shown for each patient, categorized by response to CRT. Unpaired t test p value shown. **(F)** The matched peripheral TCR clonality scores are shown for each patient at each timepoint. **(G)** The peripheral TCR clonality scores are shown for each patient at each timepoint, categorized by response to CRT. **(H)** The peripheral TCR clonal relatedness scores are shown for each patient at each timepoint, categorized by response to CRT. Mixed-effect analysis with Sidak’s multiple comparisons p values are shown for figures **F-H**. Graph bars show the median and 95%CI.

Cohort-wide, the median number of unique β-chain transcripts detected in tumor and blood samples was 8,820 and 11,200, respectively. The number of unique PBMC clonotypes was significantly lower at 6- and 12-weeks post-treatment compared to baseline, but no difference was seen between the 6- and 12-week post-treatment repertoire (**Figure 1A**). On the contrary, the absolute number of peripheral TCRs across timepoints and the proportion of unique clonotypes in relation to the total number of TCRs detected per sample across timepoints were not significantly different (**Figures 1B, C**). The total number of both, intra-tumoral detected TCRs and unique T-cells were significantly higher in HNSCC compared to CC and ASCC, as expected from a mucosa-associated lymphoid tissue (**Supplementary Figures 1A, B**). However, the proportion of unique clonotypes in relation to the total number of TCRs retrieved per sample was not significantly different across tumour types (**Supplementary Figure 1C**). On the other hand, the number of unique clonotypes and detected TCRs in peripheral blood was similar across tumor entities (**Supplementary Figures 1D, E**). In order to assess potential biases stemming from fluctuation in clonotype number between different timepoints, which might influence the metric employed for the measurement of TCR clonality, the correlation between the 1- (normalized Shannon index) and the unique number of clonotypes was explored. However, no discernible correlation was ascertained between these variables (**Supplementary Figure 1F**). Thus, this was the metric used to quantify TCR clonality, where low scores correlate with a more diverse repertoire and higher scores with an expansion of dominant TCR clones. Overall, TCR clonality in tumor samples was similar compared to baseline peripheral blood (**Figure 1D**). Intra-tumoral TCR clonality was similar across the three tumor types (**Supplementary Figure 1G**) and we only found a significant higher peripheral clonality at baseline in ASCC compared to CC patients (**Supplementary Figure 1H**). We observed higher baseline intra-tumoral TCR clonality in non-responders compared to responders (**Figure 1E**), but peripheral TCR clonality was not associated with response at any of the sampled timepoints (**Supplementary Figure 1I**). The peripheral repertoire clonality significantly increased from baseline after CRT (**Figure 1F**). However, when patients were split according to response, only responders displayed a significant increase in peripheral TCR clonality 6 weeks post-treatment compared to baseline (**Figure 1G**). Next, we computed an intra-repertoire similarity score or clonal relatedness as a metric that takes into account sequence similarity without regard for clonal frequency, to evaluate the link between response to CRT and changes in the intra-repertoire peripheral clonotype similarity. We observed that only responders displayed a significant increase in clonal relatedness at 6 weeks compared to baseline (**Figure 1H**).

### 3.3 Early post-treatment expansion of previously expanded peripheral baseline clonotypes is associated with response to CRT

**Figure 2:**
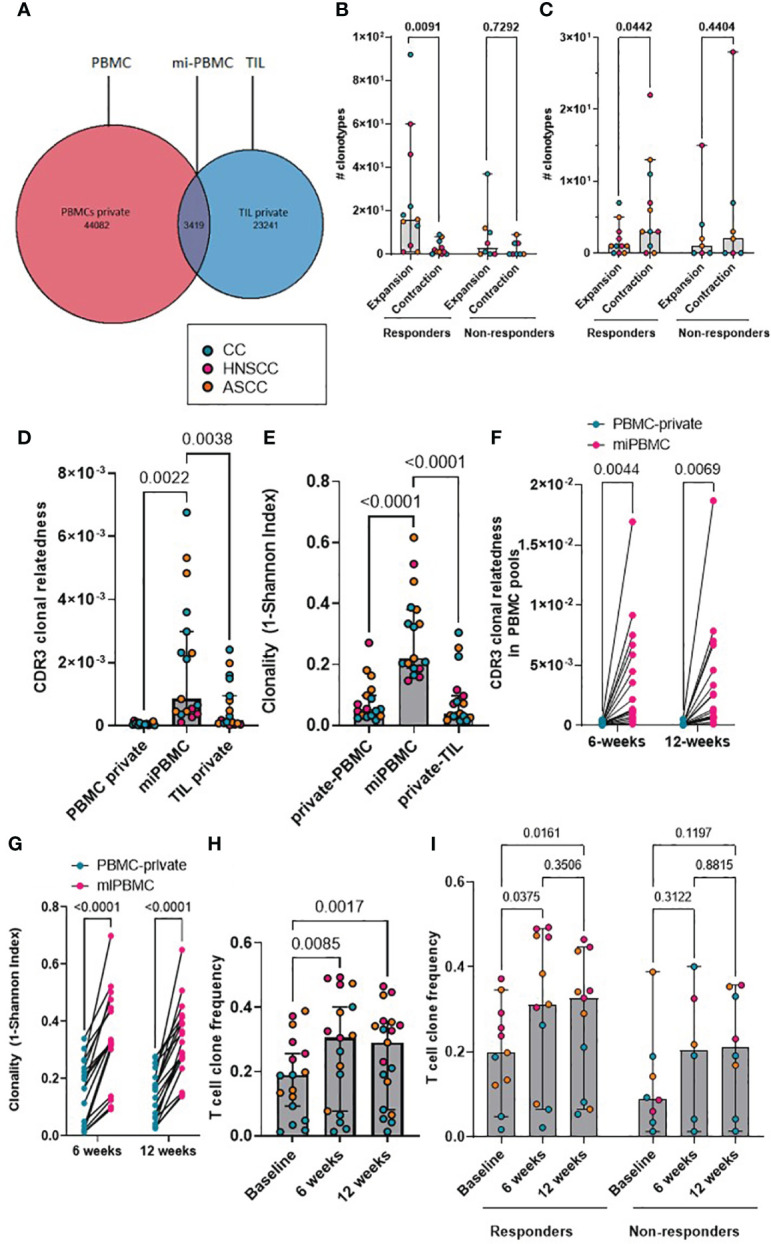
**(A)** Representative Venn diagram of a showing CDR3 sequences in PBMCs and TILs for patient HN54 (responder) at baseline. **(B, C)** Absolute number of expanded and contracted peripheral clonotypes of pre-expanded baseline clonotypes at 6 weeks **(B)** and 12 weeks **(C)** are shown for each patient categorized by response. Two-way ANOVA with Sidak’s multiple comparison p values are shown. **(D)** Clonal relatedness (proportion of CDR3β amino acid sequences that were related by a maximum edit distance of 3) in the PBMC-private, mi-PBMC and TIL-private pools at baseline. **(E)** Clonality (1-normalised Shannon index) in the PBMC-private, mi-PBMC and TIL-private pools at baseline. One-way ANOVA with Dunnet’s multiple comparisons test p values are shown for figured D and E. **(F, G)** Comparison of clonal relatedness (maximum edit distance = 3 amino acids) **(F)** and clonality (1-normalised Shannon index) **(G)** between the PBMC-private and the mi-PBMC pools at 6 and 12 weeks. Paired t-test p values are shown. **(H)** T-cell clone frequency in mi-PBMC pool at 6 and 12 weeks. Mixed-effect analysis with Sidak’s multiple comparisons p values are shown. **(I)** Comparison T cell clone frequency in mi-PBMC pool across timepoints by response. Mixed-effect analysis with Tukey’s multiple comparison corrected p values are shown. Bar graphs show median and 95% CI.

We next focused on the proportion of tumor-resident T-cell clones also present in the periphery. We classified CDR3β amino acid sequences as ‘private’ if they were only found in either tumor (TIL-private) or in peripheral blood (PBMC-private), or as ‘migrated’ PBMCs (mi-PBMCs) if they were shared between tumor and peripheral blood. As an example, at baseline, patient HN54 (responder) presented 47,501 unique CDR3β amino acid sequences in bulk PBMCs, 26,660 in TILs and 3,419 migrated sequences (**Figure 2A**). We computed the absolute number of mi-PBMCs that displayed statistically significant increase (“expansion”) or decrease in frequency (“contraction”) between two timepoints (baseline vs. 6 weeks post-treatment, and 6 vs. 12 weeks) using Fisher exact test with FDR< 0.01. We found that responders displayed a significantly higher absolute number of expanded than contracted mi-PBMCs at 6 weeks, and significantly more contracted than expanded mi-PBMCs at 12 weeks. In contrast, non-responders did not show any statistically significant difference between expanded and contracted mi-PBMCs at any timepoint (**Figures 2B, C**). In addition, subsequent contraction of previously expanded T-cell clones 12 weeks after completion of treatment was directly associated with response to treatment.

### 3.4 CRT increases peripheral TCR divergence and repertoire similarity correlates with response

We compared the clonal relatedness of the CDR3β sequences from the mi-PBMCs with the PBMC-private and TIL-private pools. At baseline, the mi-PBMCs displayed more clonal relatedness and higher clonality than both the PBMC-private and TIL-private CDR3β regions (**Figures 2D, E**), suggesting more TCR convergence and expansion in tumor-associated T cells that have potentially migrated into or from the peripheral blood, than in the bulk PBMC and TIL populations. Moreover, we consistently observed significantly higher CDR3β clonal relatedness and clonality in the mi-PBMC, compared to the PBMC-private pool, at 6 and 12 weeks post-treatment (**Figures 2F, G**). Next, we followed the mi-PBMC pools for each patient and, when comparing the relative frequency of T-cell clones across timepoints in all patients, we observed a significant increase in the proportion of the peripheral PBMC repertoire occupied by this mi-PBMC pool compared to baseline at 6 weeks and 12 weeks (**Figure 2H**). Critically however, this increase was only found to be statistically significant in the responder group (**Figure 2I**). In addition, when we classified patients by response, we only found a significant increase in the clonal relatedness of both the mi- and the private-PBMC pools in the responder group (**Supplementary Figure 2A**). Given these findings, we calculated the Morisita index as a measure of how similar pre-treatment intra-tumoral and bulk peripheral TCR repertoires were across different timepoints. The Morisita overlap index considers the relative frequency of different TCRs in two samples and it is widely used as a highly efficient estimator of dispersion. Thus, this also allowed us to evaluate the link between response to CRT and maintenance of pre-existing, or replacement with novel, TCR clonotypes. Tracking the bulk TCR pools, we observed a greater degree of TCR clonal maintenance in responders (greater TCR repertoire overlap between baseline and 12-week peripheral and intra-tumoral repertoires), compared to non-responders (**Supplementary Figures 2B-D**).

### 3.5 Pre-treatment intra-tumoral and post-treatment peripheral cluster structures are associated with response to CRT

**Figure 3:**
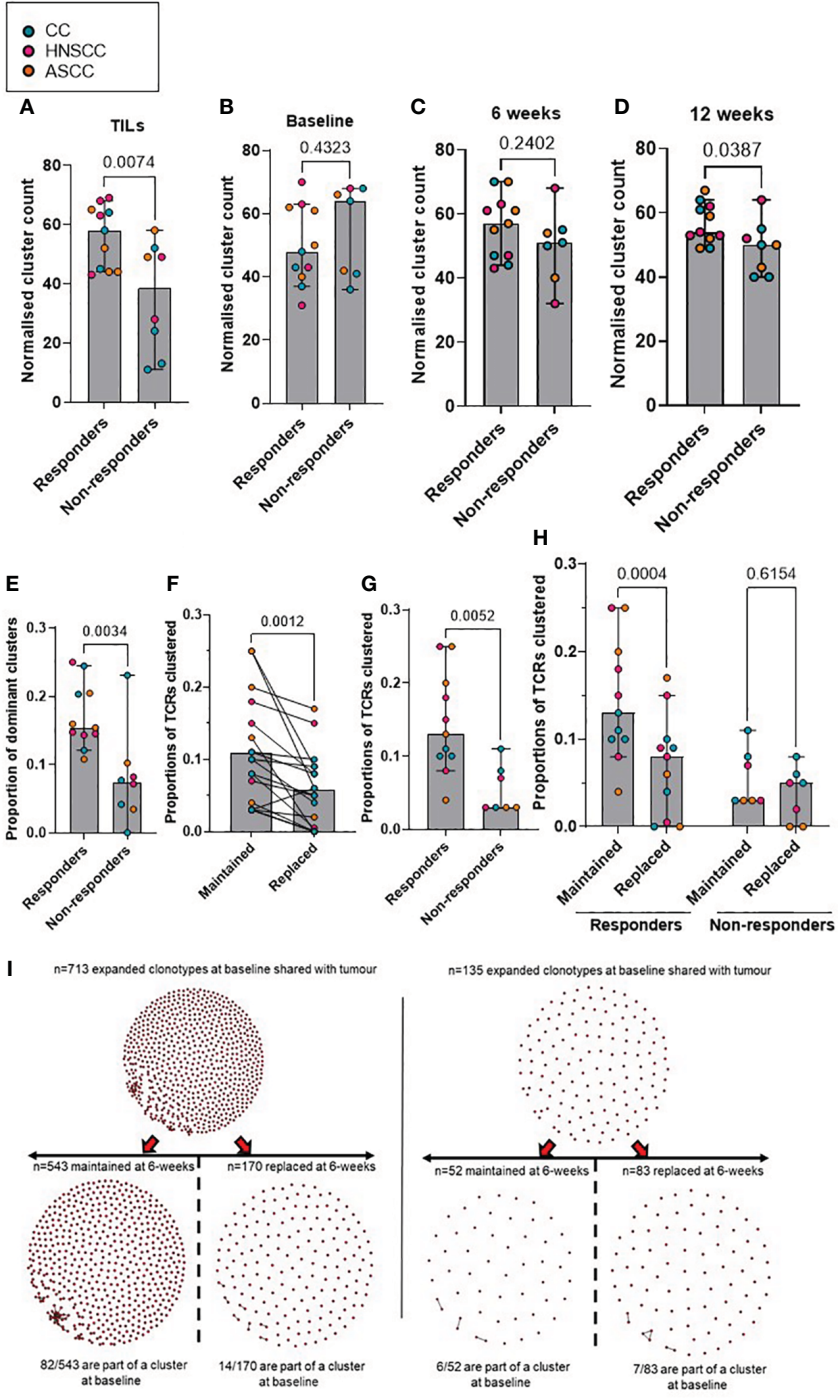
**(A–D)** Comparison according to response of normalised cluster count by CDR3β similarity for the networks containing expanded sequences in the intra-tumoral **(A)**, peripheral baseline **(B)**, 6 weeks **(C)** and 12 weeks **(D)** TCR repertoires. **(E)** Proportion of dominant TCR clusters in the intra-tumoural expanded repertoire according to response. Unpaired t test p values are shown for figures A-E. **(F)** Proportion of maintained versus replaced expanded clustered TCRs at 6 weeks from baseline which are present in the intra-tumoral repertoire. Paired t test p value is shown. **(G)** Proportion of baseline clustered structures of maintained TCRs at 6 weeks categorized by response to CRT. Unpaired t test p value is shown. **(H)** Proportion of maintained and replaced baseline clustered structured TCRs at 6-weeks categorized by response to CRT. Two-way ANOVA adjusted p values using Sidak’s multiple comparison test are shown. **(I)**. Representative kernel network diagrams for peripheral baseline and 6-weeks CDR3β-chain sequences for a responder (left) and a non-responder (right). Clustering performed within the bulk TCR sequencing data around expanded TCRs, subdivided between clones that were maintained in the 6 weeks post-treatment (left) repertoire and clones that were replaced (right). Each dot represents an expanded T-cell clone and the vectors joining each dot represent a cluster of T-cell clones. Bar graphs show median and 95% CI.

Antigen-specific T-cell responses are often associated with the presence of clusters of TCRs with similar CDR3β peptide binding sequences. As previously described, we defined expanded TCRs as those present above a threshold frequency of 2/1,000, corresponding to the top 1% of the empirical TCR frequency distribution (11), and we performed clonotype clustering analysis in the expanded tumor-resident and peripheral TCR repertoires (**Supplementary Figure 3**). We observed that expanded TCR clones showed significantly increased clustering of similar CDR3β sequences (or “cluster structures”) in responders compared with non-responders, both in the pre-treatment tumor-resident repertoire and in the post-treatment peripheral 12-week (but not earlier) repertoires (**Figures 3A-D**). In addition, when analyzing the proportion of intra-tumoral dominant clusters (where a dominant cluster was considered if it was formed by > 3 TCRs), we found a significantly higher proportion of them in the responder group, compared to non-responders (**Figure 3E**). These results are in line with the finding of a significantly higher clonal relatedness in the expanded tumor-resident TCR repertoire in the responder group compared to the non-responders (**Supplementary Figure 4A**). Next, we classified expanded TCRs in either maintained (if they were still expanded in the following timepoint) or replaced (if they were either not detected or not expanded in the following timepoint) and we applied same clustering methods (**Figure 3I**). We found that at baseline, expanded TCRs that were maintained at 6 weeks displayed significantly more clustered structures than expanded TCRs that were present at baseline and subsequently replaced (**Figure 3F**). When analyzing patients by response to CRT, we observed that responders showed significantly more baseline clustered TCR structures that were maintained at 6 weeks, compared to non-responders (**Figure 3G**). Moreover, only responders displayed statistically significantly more maintained than replaced clustered structures (**Figure 3H**). Similar results were found when comparing the 6- and 12-week expanded TCR repertoires: a significantly greater proportion of maintained, rather than replaced, clustered structures that were present at 6 weeks were still detected at 12 weeks, although this was only observed in responding patients (**Supplementary Figures 4B-D**).

### 3.6 Clustering peripheral T cell repertoire by antigen specificity using in silico models

**Figure 4:**
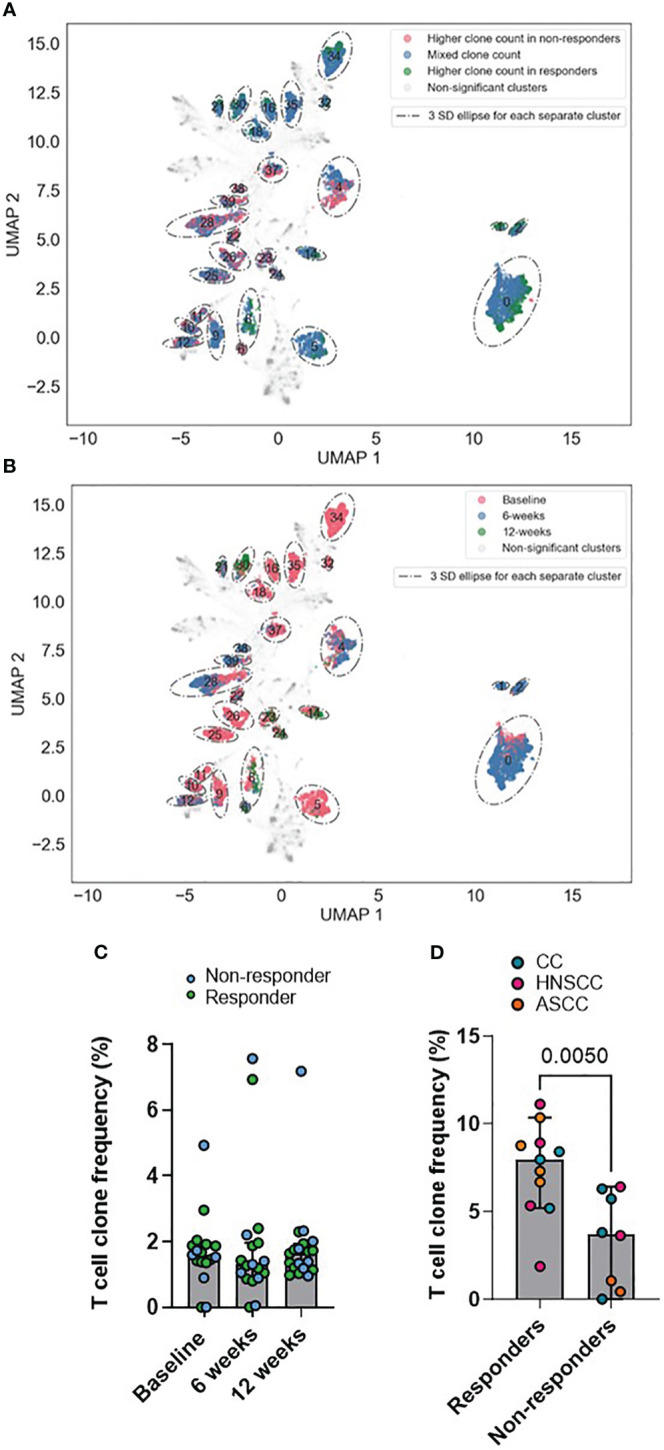
**(A, B)** UMAPs of GLIPH2 convergence groups by response **(A)** and by timepoint **(B)**. Based on the UMAP components, HDBSCAN was used to cluster the GLIPH2 clusters. For each HDBSCAN cluster, the distributions of the scaled counts were compared between responders and non-responders. Any clusters with a FDR q-value of< 0.01 is shown circled in the graph (it is a 3 SDs ellipse, based on all the points in the cluster). In A, those points belonging to a significant HDBSCAN cluster are also coloured by if the GLIPH2 convergence groups in question has a 4x higher clone count in responders, non-responders, or if the cluster is more mixed. In B, the points are coloured by which timepoint has the highest clone count in the GLIPH2 convergence groups, baseline, 6 or 12 weeks. **(C)** Peripheral frequency of the pools of T cell clones conforming clusters 6, 2 and 25 across all timepoints. **(D)** TIL frequency of the pools of T cell clones conforming the clusters 6, 2 and 25 in the pre-treatment biopsy. Unpaired t test p value is shown. Bar graphs show median and 95% CI.

As described previously (34), GLIPH2 identifies CDR3β sequences that are highly likely to share MHC-peptide specificities based on local motifs and/or global homology. To identify T-cells recognizing shared tumor antigens that may function as determinants of response, we applied GLIPH2 to the peripheral repertoire of all patients across all timepoints. A scaled count for each patient in each GLIPH2 convergence group containing sequences from more than 10 samples (n=30,222) was used as input to the UMAP and, based on the UMAP components, HDBSCAN was used to cluster the GLIPH2 convergence groups. For each HDBSCAN cluster, the distributions of the scaled counts were compared between responders and non-responders (**Figure 4A**) and across timepoints (baseline, 6 and 12 weeks) (**Figure 4B**). Interestingly, we found some clusters with significantly increased counts in either responders (i.e. clusters 1 and 2) or non-responders (i.e. clusters 6 and 39). In addition, despite most of the clusters showing a significantly higher count at baseline, some of them were more frequently represented at 6 weeks (i.e. clusters 1 and 2) and 12 weeks (i.e. clusters 21 and 30). Next, we used a previously described in silico pipeline for prediction of specific CDR3β amino acid sequences and peptide binding from large dictionaries of TCR-peptide pairs (ERGO) to predict the binding probability between the CDR3β that form each cluster and HPV16 peptides (37). Eight to 11-mer HPV16 peptides, derived from the E2, E5, E6 and E7 viral oncoproteins, were selected according to their percentage elution rank (equal or lower than 1) as strong binders according to the HLA type of each individual patient (**Supplementary Table 1**) using netMHCpan-4.1. Comparing the pooled TCR-peptide binding probabilities for each HDBSCAN cluster with all the others, we found that 3 clusters showed significantly higher HPV16-specific binding probability, whereas 6 clusters displayed significantly lower binding probability (**Supplementary Table 2**). We, therefore, focused on these 3 HPV16-specific TCR clusters (6, 2 and 25), and calculated their frequency in the TILs and PBMCs. We found that, despite the predicted HPV16-specific clones displaying similar relative abundance across all timepoints (**Figure 4C**), there was a significantly higher frequency in the pre-treatment TILs of the responder group (**Figure 4D**). These findings suggest that in the responding group there was a higher proportion of HPV16-specific T cells in the tumor prior to treatment, although the anti-HPV16 peripheral clones were equally abundant regardless of response.

## 4 Discussion

We present results of a translational study with sequential TCR analysis of samples from patients with locally advanced HPV16-driven malignancies treated with CRT. To our knowledge this is the first study investigating spatial (tumor versus peripheral) and temporal changes in T-cell repertoire to predict treatment outcomes in this setting. This study was proposed in the assumption that mean clonality would be different according to response to treatment. Although this was confirmed in the intra-tumoral TCR repertoire, the clonality index was lower than previously expected (mean clonality and SD difference of 0.07 and 0.06, respectively). This will serve for subsequent studies interrogating the TCR repertoire in this set of patients.

Some patterns of peripheral repertoire turnover (such as changes in the clonality and clonal relatedness) have been associated with an effective immune awakening in patients treated with immune checkpoint inhibitors (ICIs) (6–8, 38, 39). Our data identify some baseline intra-tumoral characteristics that may drive response to treatment, such as TIL high diversity and increased TIL clustering in the pre-treatment biopsy. The debate regarding the primary mechanism underlying ICI response focuses on two hypotheses: “T-cell clonal replacement,” which involves the recruitment of novel T-cells into the tumor, and “T-cell clonal revival,” which suggests the reinvigoration of pre-existing TILs (40). Clonal replacement mechanism has been supported in studies that included patients with skin cancer and NSCLC which showed that anti-PD-1 therapy promotes the infiltration of T-cells from the blood into the tumor by inducing recognition of neoantigens different from those recognized by expanded TCR clones at baseline, and that patients with major pathological response showed substantial overlap between intra-tumoral and peripheral blood TCR clonotypes, with most expanded clones shared between the two compartments (41, 42). An alternative perspective (clonal revival) suggests that ICI response arises from pre-existing intra-tumoral T-cells that are primed by neoantigens and capable of cytotoxic reinvigoration despite exhibiting exhaustion features. This has been supported in a recent study involving patients with metastatic renal clear-cell carcinoma treated with anti-PD-1 therapy which showed that responders had higher pre-treatment intra-tumoral TCR clonality and cluster structure compared to non-responders, indicating the presence of pre-existing adaptive immunity (7). Accordingly, our data show that the proportion of shared TIL-PBMC clonotypes increases with treatment, and that the responder group displays higher overlap between TIL and PBMCs and increased baseline intra-tumoral diversity and clustered structures than the non-responder group. To reconcile the seemingly conflicting reports supporting either clonal replacement or clonal revival, it is essential to consider that the mechanisms underlying ICIs response may be context-dependent and can vary among different malignancies, tumor samples (primary versus metastatic), on-treatment sampling timings, ICIs regimes and dosage. There is paucity of data regarding the effects that CRT has in the intra-tumoral and peripheral TCR repertoire and there is a high chance that these will be different to the ICI effects and will vary according to the irradiated site, the dose and the use of concurrent cytotoxic drugs. Our results point towards the fact that the expansion of tumor-infiltrating T-cell clones secondary to CRT is probably driven by pre-existing expanded and antigen-specific TILs which can be found and tracked in the peripheral blood, where they display different dynamics according to response to treatment. In this regard, we demonstrated that there is a population of peripheral expanded pre-treatment TCR clonotypes that are shared with tumor and are preferentially maintained and/or expanded by CRT, potentially reflecting enhanced stimulation by increased antigen cross-presentation and priming, and/or the ability of CRT to promote the activation and expansion of T-cells which are already primed. These findings, whilst not previously demonstrated in patients treated with CRT, have been described in longitudinal samples of patients treated with ICIs and, importantly, support a role for anti-tumor immunity in the response to CRT as currently used in the clinic (7). In addition, this paradigm has long been supported by pre-clinical murine data, but not by robust clinical evidence (43). Although the precise reason for the drop in their relative abundance 12-weeks following completion of CRT in the responder groups unknown, it may reflect that the resolution of active disease following therapy reduces the potential source of antigen required to maintain cancer-specific T cell immunity. However, another explanation for this may be sequestration of tumor-antigen reactive T cells from peripheral blood to the tumor. Indeed, this notion has been recently shown in a cohort of melanoma patients treated with anti-PD-1 therapy (44) and supported by Bhatt et al., who made the interesting observation that specific CD8+ and CD4+ anti-HPV T-cell reactivity dropped after radical CRT (45). On the other hand, the absence of either expansion or contraction of baseline TCR clones in non-responders reflects a failure of activation of effector anti-tumor T-cells during CRT, potentially associated with maintained presence of tumor antigens 12 weeks after completion of treatment.

The concept of mi-PBMCs allowed us to follow T cells that are shared between peripheral blood and tumor and that are presumed to have an anti-cancer immune function in the tumor microenvironment. The significant increase in the clonal relatedness of both the mi- and the private-PBMC pools in the responder group suggests a more pronounced reconfiguration in the peripheral TCR repertoire in responding, as opposed to non-responding, patients. Furthermore, our results imply that CRT induces TCR repertoire divergence, with an increase in the shared TIL-PBMC repertoire (mi-PBMC) frequency after treatment only in responders. Moreover, there is also a broader compartmental overlap (increased Morisita index between baseline tumor-resident and peripheral T-cell repertoire) and longitudinal sharing (increased clonal relatedness of mi-PBMCs) in the TCR repertoire in patients who responded.

Maintenance of early post-treatment (6 weeks) expanded clustered structures only in responders suggests that clonal preservation of pre-expanded T cells that share epitope-specificity functions as a pre-requisite of response to treatment, whereas loss or replacement of T cell clusters may reflect an ineffective anti-cancer immune response or tumor-induced tolerance in patients who do not respond. Furthermore, there is a population of closely related TCR clonotypes in responders that are expanded pre-treatment and detected in both tumor and peripheral blood and that these are preferentially maintained and/or expanded by CRT. This may reflect enhanced stimulation by increased antigen cross-presentation and T-cell priming, and/or the ability of CRT to promote primed T-cell activation and expansion.

Interestingly, our results also show that responding patients display similar predicted HPV16-specific peripheral T cell abundance to the non-responding patients, but a higher proportion of these in the TIL compartment pre-treatment. Moreover, although these tumor-infiltrating clonotypes show lower pre-treatment clonality in the responder group, they display increased clustering capacity, which suggests that a richer antigen-shared T cell repertoire is a key element of an effective anti-cancer immune response.

Our study sheds light on the determinants of response to standard-of-care CRT, and in particular the nature of the adaptive T cell response likely contributing to anti-tumor immunity. Despite the increased number of studies that have investigated possible predictive biomarkers of response to CRT in different HPV-driven malignancies, currently there are no markers of response used as standard-of-care in clinical practice (46). To date, most predictive markers of response have focused on the evaluation of pre-treatment tumor tissue. However, given the challenge of performing serial tissue biopsies during CRT, it is necessary to develop alternative strategies, that are reproducible and minimally invasive, to allow real-time monitoring of the clinical (and immune) response and disease evolution throughout treatment. In this regard, biomarkers such as circulating HPV DNA, or TCR repertoire analysis, can potentially be used for monitoring disease response during and following radical CRT (29, 47).

There are limitations to our study. First, the small number of patients limits data generalizability, and findings from this study would benefit from validation in larger datasets. We acknowledge that the differing group size, with smaller sample size in the non-responder group, suggests caution in missing an effect due to reduced power. However, our scope for translational discovery was supported by the extent of longitudinal tracking and in-depth analysis of the TCR repertoire changes under therapy. We acknowledge the fact that although all patients included in this study were diagnosed with locally-advanced HPV16-driven malignancies, they belong to three different tumor entities. Moreover, although the cornerstone treatment for all these three malignancies is radiotherapy, the total dose, fractionation, and concurrent chemotherapy agent used was different according to the tumor type. The potential influence of concurrent mitomycin C used for the treatment ASCC compared to cisplatin (used in HNSCC and CC) in the peripheral TCR repertoire is unknown. However, there is evidence that both cisplatin and mitomycin C are relatively inefficient in stimulating immunogenic cell death in the absence of radiation (48). Thus, one chemotherapy agent is not expected to be more immunogenic than the other. Since HPV genotype impacts the prognosis and response to CRT, especially in CC, only patients with confirmed HPV16 genotype (and no evidence of other HPV types) were included in this study. In addition, although these three entities display different biology, there is not enough evidence to assert that they display different TCR dynamics. Indeed, a recently published study which included cervical, vaginal, vulvar and anal carcinomas did not find differences in the antigen-specific immune response to CRT across these tumor types (49). Our results are consistent with this investigation and with the effects of radiation observed in preclinical models and human case reports across different types of malignancies (9, 50, 51). Although further studies are required to determine the mechanisms underpinning our observations and their specificity for CRT-induced responses, our research functions as a hypothesis generating study and provides a strategy to analyze immune cell evolution in patients treated with radiotherapy, chemotherapy, ICIs, or their combinations. This methodological strategy includes a novel pipeline which integrates the output from different and validated in silico predictions for MHC presentation, TCR clustering and TCR-epitope binding. We acknowledge that the use of TCR clonality metrics alone does not provide a definitive assessment of anti-cancer T-cell responses. However, analysis TCR sequence similarity in the form of clusters (grouping TCRs with similar CDR3 peptide sequences) and networks (grouping closely related TCR clusters) provides insights into the antigenic specificity of TCRs. Indeed, in a recent analysis of TCR sequencing metrics from the TRACERX consortium which combined multi-region tumor samples and adjacent non-tumor tissue obtained from patients with early-stage NSCLC, authors showed the presence of clusters and networks of related TCR sequences within populations of TILs (11). Furthermore, whilst we acknowledge the limitations of the in silico strategies, they provide a first step towards a better understanding of the immune repertoire and its dynamics. Indeed, the proportion of our predicted HPV16-specific clonotypes in peripheral blood and tumor are consistent with previous publications (16). Looking forward, multiparameter flow cytometry, circulating tumor and HPV16 DNA, transcriptomic profiling techniques (including single cell) and additional ex vivo functional immune experiments to track T cell responses to HPV16 peptides, will be invaluable in studying baseline tumor microenvironment and peripheral immune changes to treatment. In this regard, previously published studies have interrogated the dynamics of circulating tumor and HPV16 DNA in plasma and the HPV viral load in tumor in HPV-driven malignancies treated with radical radiotherapy or CRT as an approach to predict disease progression and survival (29, 47, 52, 53).

In summary, in this translational study, we identified intra-tumoral and peripheral TCR metrics and in silico HPV16-specific T cell clones which hold promise for use as predictive markers of response to radical CRT and provide a template for future larger cohort immune-oncology biomarker studies in HPV-driven malignancies. Our findings advance the knowledge of immune responses to CRT and, critically, provide a potentially tractable tool to identify which patients will not respond or relapse early following radical CRT. This could eventually help clinicians to stratify patients more effectively and to consider poor-responder patients for adjuvant immunotherapeutic or other additional approaches, thereby improving personalization of therapeutic planning.

## Data availability statement

TCR sequencing and HLA-typing datasets supporting the conclusions of this article are available in the figshare repository (DOI: 10.6084/m9.figshare.6025748.v1).

## Ethics statement

The studies involving humans were approved by Institutional board and ethics committee 14/NE/1055. The studies were conducted in accordance with the local legislation and institutional requirements. The participants provided their written informed consent to participate in this study.

## Author contributions

PN: Data curation, Formal analysis, Investigation, Methodology, Visualization, Writing – original draft, Writing – review & editing. AL: Formal analysis, Methodology, Writing – review & editing. FM: Formal analysis, Investigation, Methodology, Writing – review & editing. JL: Data curation, Formal analysis, Investigation, Methodology, Writing – review & editing. SL: Investigation, Writing – review & editing. DG: Investigation, Writing – review & editing. MP: Methodology, Software, Writing – review & editing. B’OL: Writing – review & editing. AR: Writing – review & editing. AS: Methodology, Writing – review & editing. BC: Formal analysis, Methodology, Resources, Writing – review & editing. AM: Supervision, Writing – review & editing. KH: Funding acquisition, Supervision, Writing – review & editing. SB: Conceptualization, Funding acquisition, Supervision, Writing – review & editing.
