# Supplementary material for: T-cell receptor determinants of response to chemoradiation in locally-advanced HPV16-driven malignancies
Source: Front Oncol. 2024 Jan 3;13:1296948. doi: 10.3389/fonc.2023.1296948 (PMC10791873; doi:10.3389/fonc.2023.1296948)
Supplement: Supplementary file 1 [file DataSheet_1.pdf]

Supplementary materials

Table of contents:

- Supplementary Figure 1.
- Supplementary Figure 2.
- Supplementary Figure 3.
- Supplementary Figure 4.
- Supplementary Table 2.

Supplementary Figure 1.

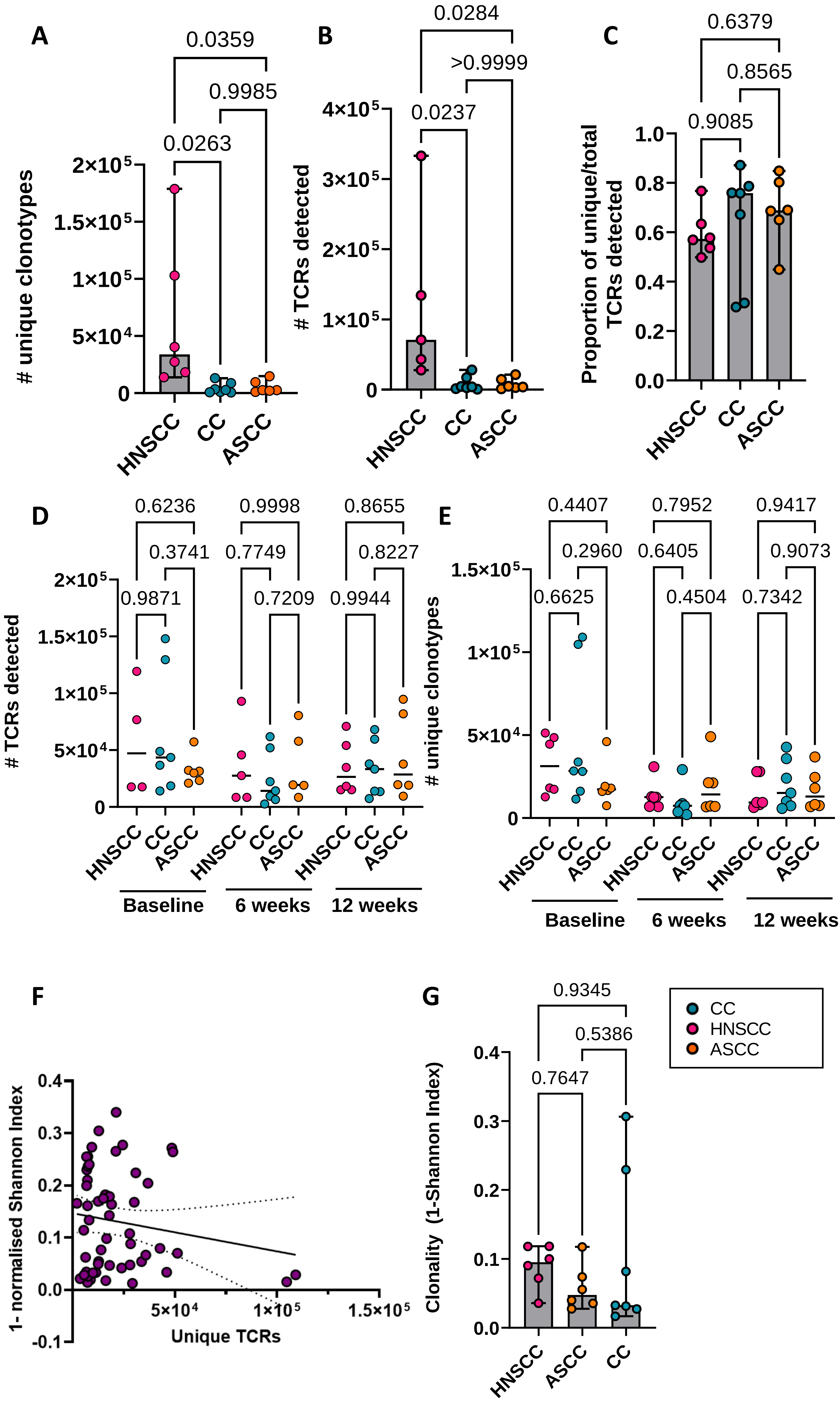

H

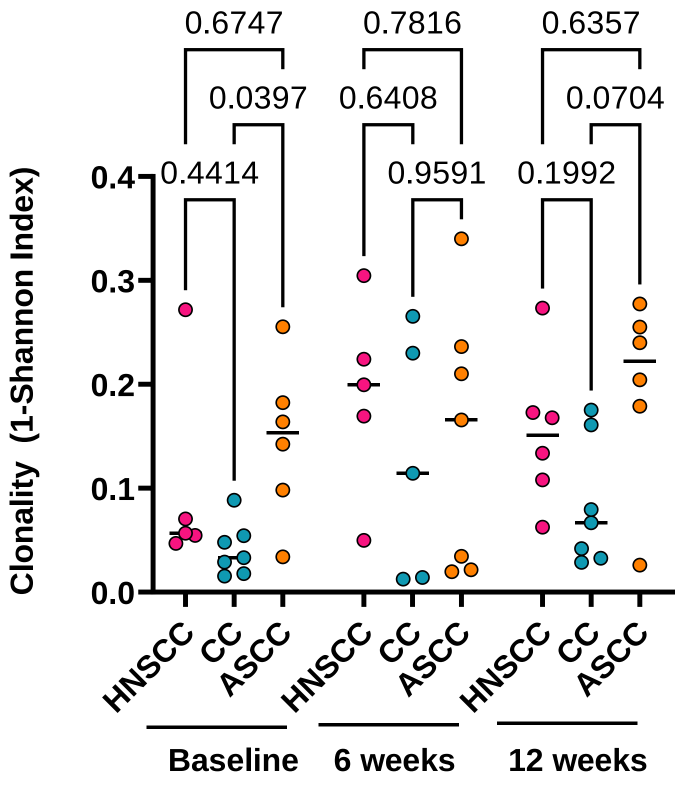

I

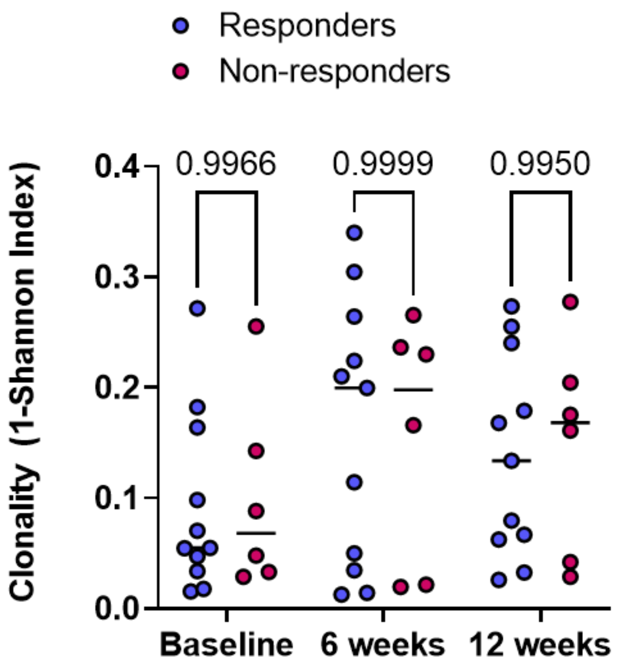

Supplementary Fig 1. (A-C) Unique number of clonotypes (a), absolute number of TCRs detected (b) and proportion of unique/total TCRs detected (c) in the intra-tumoral repertoire according to tumor entity. One way ANOVA with Tukey's multiple comparison correction p values are shown for a and b mixed effect analysis with Tukey's multiple comparison correction p values are shown for c. (D-E) Total number of peripheral total (D) and unique clonotypes (E) detected by tumor entity. One way ANOVA with Tukey's multiple comparison correction p values are shown. (F) Correlation between unique TCRs detected and 1-(normalized Shannon index). Pearson p value (two-tailed) = 0.24. (G) Clonality of intra-tumoral TCR repertoire across tumor entities. (H) Clonality of peripheral TCR repertoire across timepoints by tumor types. One way ANOVA with Tukey's multiple comparison correction p values are shown for figures G-H.. (I) Comparison of clonality of peripheral TCR repertoire according to response across each timepoint. Unpaired t-test p values are shown. Bar graphs show median and 95% CI.

Supplementary Figure 2.

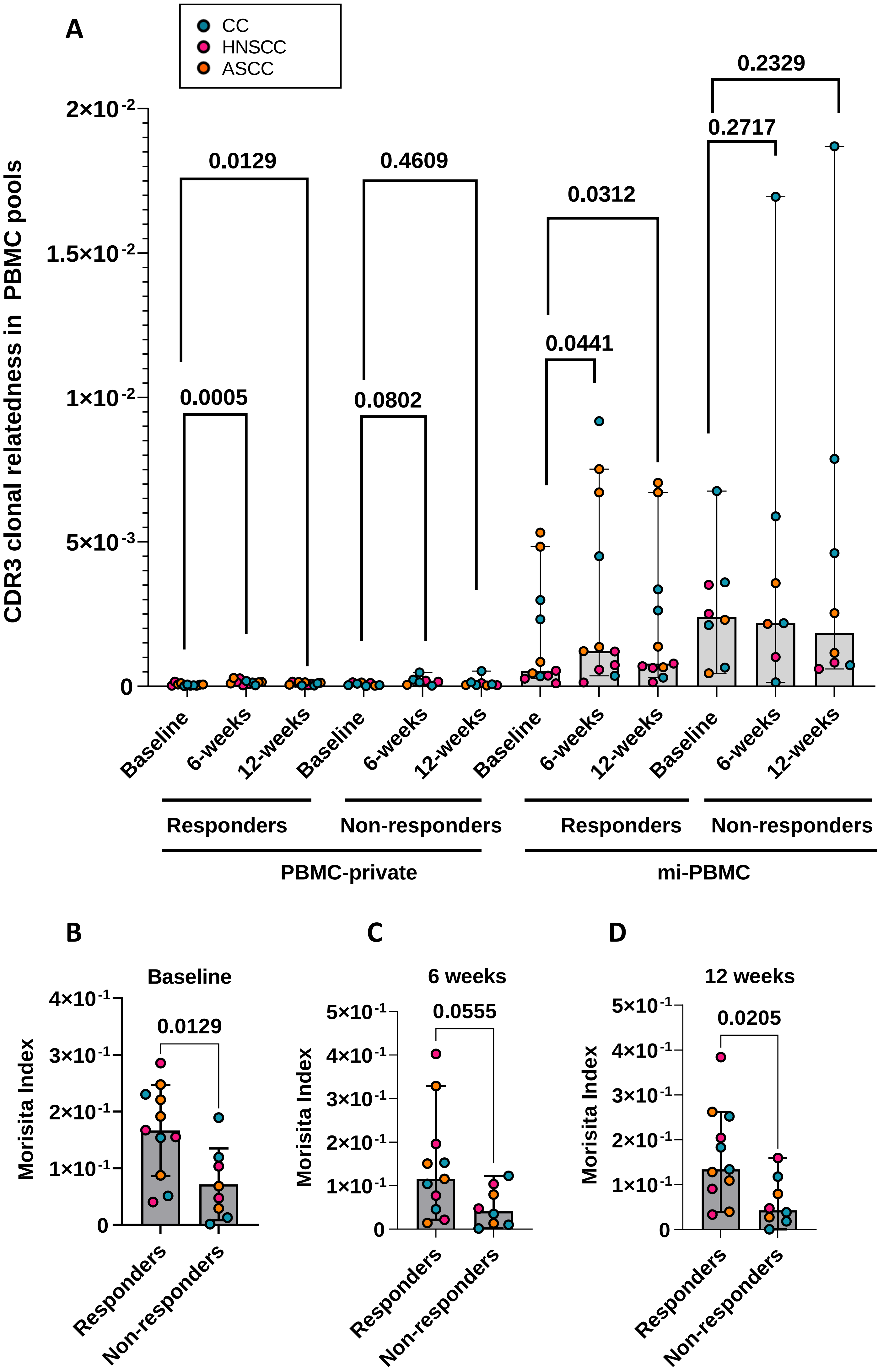

Supplementary Fig 2. (A) Clonal relatedness (maximum edit distance = 3 amino acids) for CDR3β sequences in PBMC TCR pools across timepoints. Comparisons were made separately between the baseline clonal relatedness of private-PBMC TCRs and mi-PBMCs of patients classified by response. Mixed-effect analysis with Sidak's multiple comparisons p values are shown. (B-D): Morisita index between intra-tumoural TCR repertoire and peripheral repertoire at baseline (B), 6 weeks (C), and 12 weeks (D) is shown for each patient categorized by response to CRT. Unpaired t test p values are shown. Bar graphs show median and 95% CI.

Supplementary Figure 3 (amended).

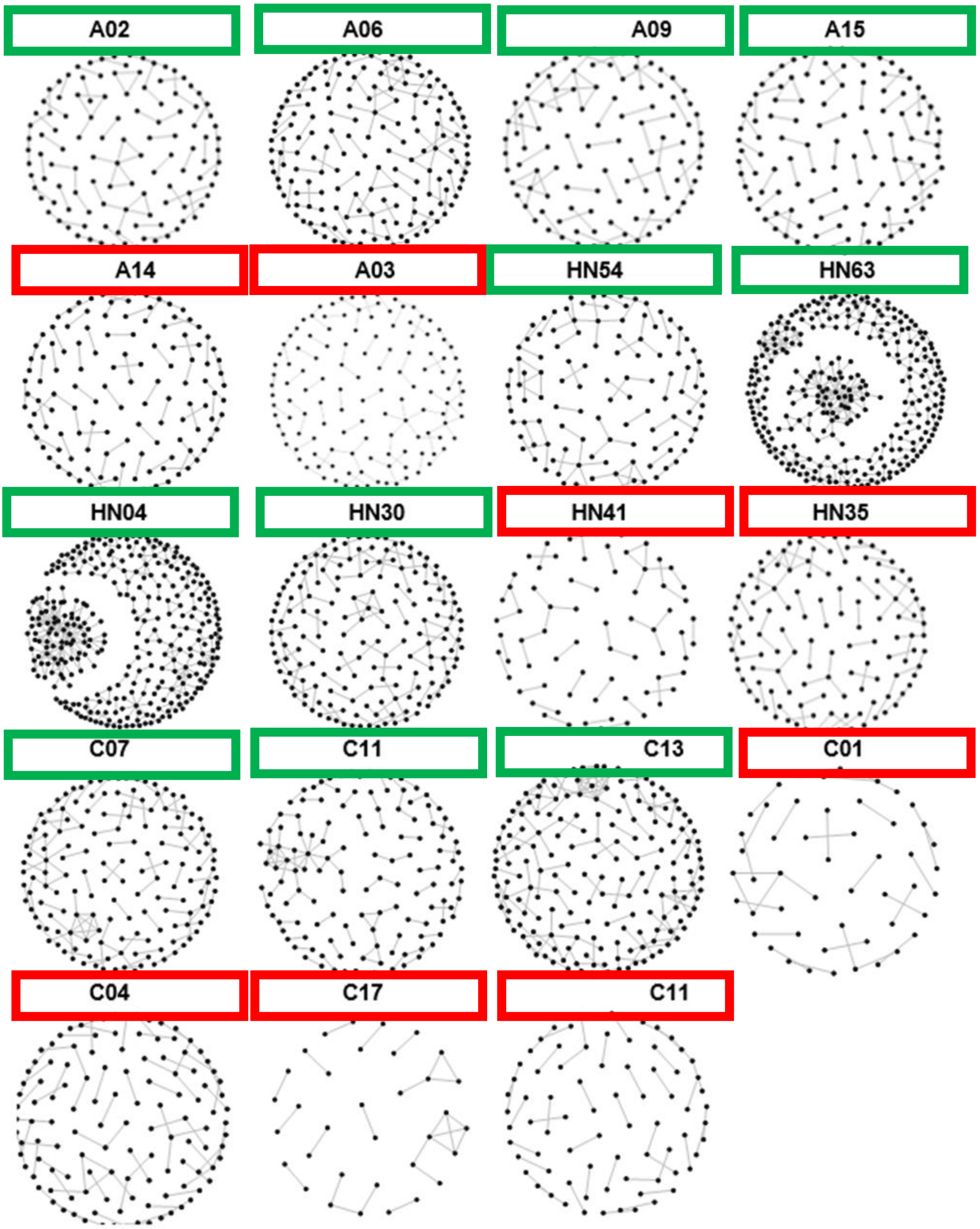

Supplementary Figure 3. Network diagrams for intra-tumoral CDR3β-chain expanded sequences for each individual patient. Green samples for responders and red samples for non-responders.

Supplementary Figure 4.

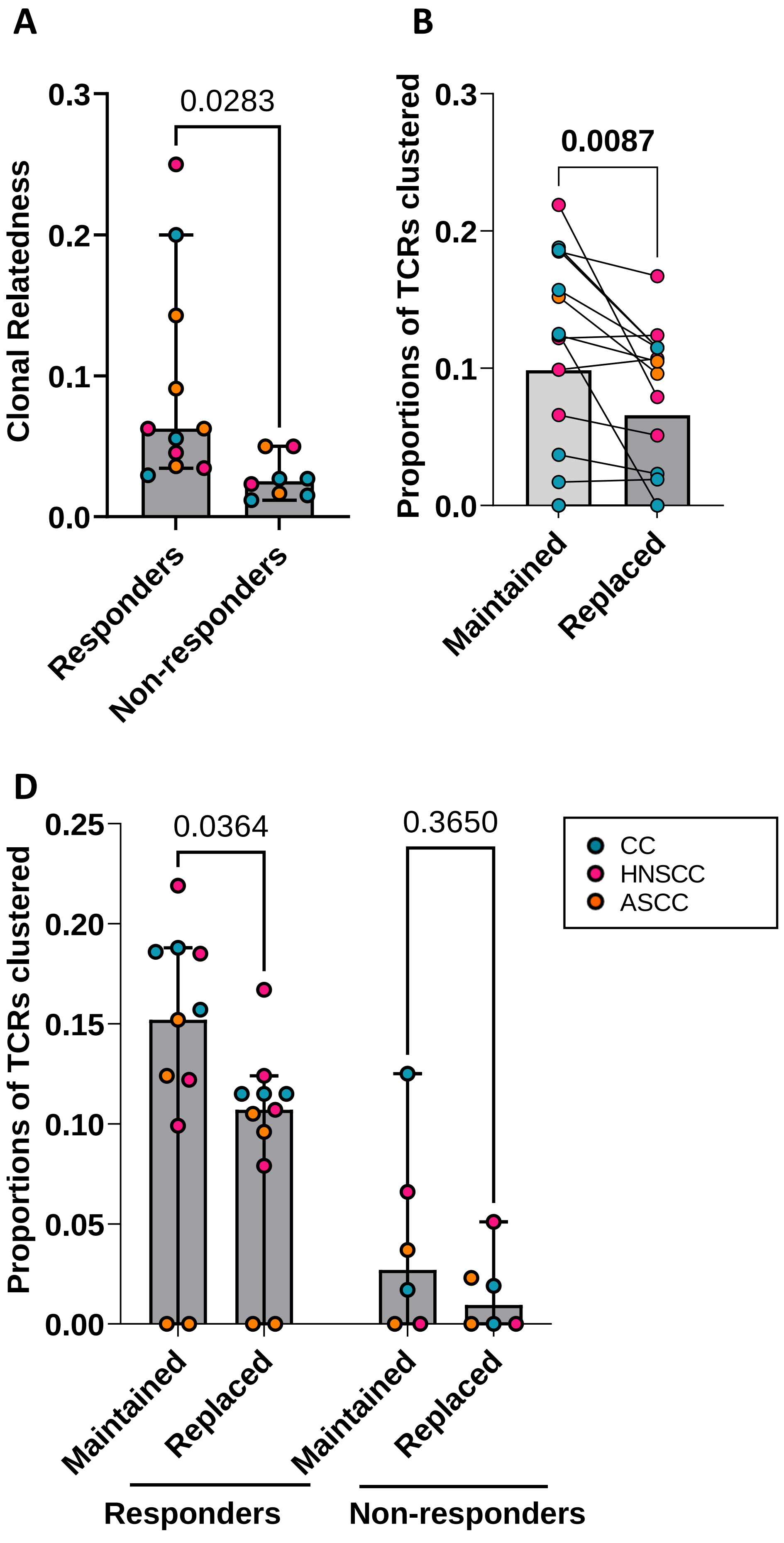

Supplementary Figure 4. (A) Clonal relatedness of intra-tumoral expanded TCR repertoire for each individual categorized by response to CRT. Unpaired t test p value is shown. (B) Proportion of maintained versus replaced expanded clustered TCRs at 12 weeks from 6 weeks which are present in the intra-tumoral repertoire. Paired t test p value is shown. (C) Proportion of baseline clustered structures of maintained TCRs at 12 weeks categorized by response to CRT. Unpaired t test p value is shown. (D) Proportion of maintained and replaced baseline clustered structured TCRs at 12 weeks categorized by response to CRT. Two-way ANOVA adjusted p values using Sidak’s multiple comparison test are shown. Bar graphs show median and 95% CI.

Supplementary Table 2.

HDBSCAN cluster	Odds ratio	Fisher p value	Adjusted p value
**UMAP_6**	0.267247	2.93E-19	8.20E-18
**UMAP_2**	0.634703	1.47E-06	2.06E-05
UMAP_8	0.713513	0.005212	0.014593
UMAP_38	0.729142	0.089903	0.11987
**UMAP_25**	0.7417	0.001539	0.004787
UMAP_37	0.773486	0.010385	0.02077
UMAP_5	0.827829	0.050415	0.074295
UMAP_35	0.877839	0.176127	0.215299
UMAP_4	0.880921	0.085988	0.11987
UMAP_0	0.889226	0.025421	0.040809
UMAP_34	0.944153	0.491859	0.510076
UMAP_28	0.952672	0.488947	0.510076
UMAP_23	1.022544	0.941947	0.941947
UMAP_14	1.202706	0.176853	0.215299
UMAP_24	1.212639	0.263575	0.307504
UMAP_32	1.248004	0.40733	0.456209
UMAP_26	1.37372	0.00798	0.020313
UMAP_9	1.375097	0.021822	0.038188
UMAP_16	1.397515	0.012196	0.022766
UMAP_18	1.409733	0.009576	0.020624
**UMAP_12**	1.541492	0.00122	0.004269
UMAP_1	1.597301	0.026234	0.040809
**UMAP_30**	1.658797	0.000373	0.001739
UMAP_22	1.76977	0.008826	0.020595
**UMAP_39**	1.874644	1.94E-05	0.000181
**UMAP_11**	1.918756	0.000754	0.003016
**UMAP_10**	1.988563	7.18E-05	0.000503
**UMAP_21**	2.144533	0.000186	0.001041
